# Acetaldehyde-ethanol interactions on calcium-activated potassium (BK) channels in pituitary tumor (GH3) cells

**DOI:** 10.3389/fnbeh.2013.00058

**Published:** 2013-06-14

**Authors:** Astrid G. Handlechner, Anton Hermann, Roman Fuchs, Thomas M. Weiger

**Affiliations:** ^1^Division of Cellular and Molecular Neurobiology, Department of Cell Biology, University of SalzburgSalzburg, Austria; ^2^Neurosignaling Unit, Department of Organismic Biology, University of SalzburgSalzburg, Austria

**Keywords:** ethanol, acetaldehyde, BK channels, GH3 pituitary tumor cells, patch-clamp technique

## Abstract

**Background:** In the central nervous system ethanol (EtOH) is metabolized to acetaldehyde (ACA) primarily by the oxidative enzyme catalase. Evidence suggests that ACA is responsible for at least some of the effects on the brain that have been attributed to EtOH. Various types of ion channels which are involved in electrical signaling are targets of EtOH like maxi calcium-activated potassium (BK) channels. BK channels exhibit various functions like action potential repolarization, blood pressure regulation, hormone secretion, or transmitter release. In most neuronal and neuroendocrine preparations at physiological intracellular calcium levels, EtOH increases BK channel activity. The simultaneous presence of ACA and EtOH reflects the physiological situation after drinking and may result in synergistic as well as antagonistic actions compared to a single application of either drug. The action of ACA on electrical activity has yet not been fully established.

**Methods:** GH3 pituitary tumor cells were used for outside-out and inside-out patch-clamp recordings of BK activity in excised patches. Unitary current amplitude, open probability and channel mean open time of BK channels were measured.

**Results:** Extracellular EtOH raised BK channel activity. In the presence of intracellular ACA this increment of BK activity was suppressed in a dose- as well as calcium-dependent manner. Mean channel open time was significantly reduced by internal ACA, whereas BK channel amplitudes were not affected. The EtOH counteracting effect of ACA was found to depend on succession of application. EtOH was prevented from activating BK channels by pre-exposure of membrane patches to ACA. In contrast BK activation by a hypotonic solution was not affected by internal ACA.

**Conclusions:** Our data suggest an inhibitory impact of ACA on BK activation by EtOH. ACA appears to interact specifically with EtOH at BK channels since intracellular ACA had no effect when BK channels were activated by hypotonicity.

## Introduction

Evidence suggests that acetaldehyde (ACA) is responsible for at least some of the effects on the brain that have been attributed to ethanol (EtOH) (Quertemont et al., 2005a). Peripheral accumulation of ACA in the blood accounts for aversion by producing unpleasant physical symptoms (Eriksson, 2001). This is different in the brain where ACA is supposed to be responsible for some rewarding and reinforcing effects of EtOH (Rodd-Henricks et al., 2002; Quertemont et al., 2005b; Karahanian et al., 2011). Importantly, EtOH oxidation has been found within the living brain revealing catalase to be the predominant enzyme responsible for ACA accumulation (Zimatkin et al., 1998; Zimatkin and Buben, 2007). The question, whether effective amounts of ACA derived from peripheral EtOH metabolism can pass through the blood-brain-barrier has, however, not been answered conclusively (Correa et al., 2011).

ACA has been shown in a few studies to modulate ion channels. For instance the action potential activity of dopaminergic neurons in the mesolimbic system is increased due to its action on IA (A-type) and Ih (hyperpolarization-activated inward) K^+^ currents (Foddai et al., 2004; Melis et al., 2007). In contrast a decrease in activity was reported for voltage-gated L-type calcium channels in neuronal cells (Bergamaschi et al., 1988) and in smooth muscle cells (Morales et al., 1997).

GH3 cells, isolated from rat pituitary tumors, are excitable neuroendocrine cells which produce growth hormone and prolactin (Tashjian et al., 1970). Further, they express calcium-activated maxi potassium channels (also referred as BK, Maxi-K_Ca_, KCNMA1, KCa1.1, or Slo1 channels) and are used in numerous studies as model cells to study BK channel properties (for review see: Weiger and Hermann, 2009; Hermann et al., 2012a). Our study focused on these channels, which are abundantly expressed throughout the body and exhibit various functions like action potential repolarization, regulation of blood pressure, hormone secretion or transmitter release (recently reviewed in Hermann et al., 2012a). BK channel activity is initiated by depolarization and enhanced by the simultaneous increase in free intracellular calcium (Ca^2+^) concentration (McManus, 1991). According to this property BK channels represent a link between the intracellular second messenger system and the electrical state of the cell membrane. BK channel activity can be altered by a wide variety of modulatory factors, including changes in pH (Church et al., 1998), redox potential (DiChiara and Reinhart, 1997), protein kinases or phosphatases (Reinhart et al., 1991; Reinhart and Levitan, 1995), interactions with auxiliary beta (β) subunits (Weiger et al., 2000), or gasotransmitters (Hermann et al., 2012b). BK channels are also involved in behavioral processes like clock controlled behavior (Montgomery et al., 2013), in behavioral responses to EtOH (Davies et al., 2003), or react to social stress with a change in expression patterns (Chatterjee et al., 2009).

BK channels as integral membrane proteins are prominent cellular targets for EtOH, which is well documented to increase BK channel activity via a Ca^2+^ and protein kinase C (PKC) dependent mechanism in a dose dependent manner. This potentiation is based on the increment of channel open probability (Po) and mean channel open time (MCOT) and thus is related to channel gating, whereas ion conductance and selectivity are not affected (Dopico et al., 1996; Jakab et al., 1997). The EtOH-mediated activation of BK channels leads to hyperpolarization of the membrane potential disposing the cell to reduce hormone secretion and transmitter release (Dopico et al., 1999). Furthermore, BK channels show fast adaptation to EtOH appearing in the form of a rapidly reduced sensitivity to acute EtOH exposure within a few minutes. This molecular tolerance is intrinsic to the channel and can be overcome by the association with a β4-subunit, an assembly often found in the brain (Martin et al., 2008). EtOH as well as hypotonicity (Hypo) induce cell swelling and both increase BK channel activity. Additionally, EtOH induced cell swelling comes along with an increment of the intracellular Ca^2+^ concentration (Jakab et al., 2006). With regard to physiological effects, EtOH modulation of BK channels influences neuronal excitability, cerebrovascular tone, brain function, and behavior (Brodie et al., 2007; Liu et al., 2008).

Basic knowledge about neurochemical mechanisms and molecular targets of ACA is poor. Little is known about the action of ACA on ion channels, including BK channels. Although EtOH and ACA are present simultaneously in brain after drinking usually each of these chemicals is investigated separately in experimental settings. In our study both drugs were applied individually, simultaneously or successively in order to reveal possible interactions.

## Materials and methods

### Cell culture

GH3 pituitary tumor cells were cultured in MEM-Eagle (Minimum Essential Medium, Sigma, Vienna, Austria), enriched with 7% fetal bovine serum and with 3% horse serum (sera from Invitrogen, Vienna, Austria). Cells were grown in tissue culture flasks at 37°C, 95% humidity and 5% CO_2_ and fed two times a week. For electrophysiological recordings cells were seeded on PDL (poly-D-lysine, Sigma, Vienna, Austria) coated glass cover slips and used after 2–5 days for experiments. Cell passages 10–60 (internal count) were used in this study.

### Solutions

All chemicals were from Sigma (Vienna, Austria). Bath solution (mM): 145 NaCl, 5 KCl, 1 MgCl_2_, 1 CaCl_2_, 10 HEPES, 10 glucose; EtOH solution isoosmolar (mM): 130 NaCl, 5 KCl, 1 MgCl_2_, 1 CaCl_2_, 10 HEPES, 10 glucose, 30 EtOH; 30% hypotonic solution (mM): 110 NaCl, 5 KCl, 1 MgCl_2_, 1 CaCl_2_, 10 HEPES, 10 glucose; standard pipette solution (mM): 140 KCl, 2 MgCl_2_, 0.88 CaCl_2_, 1 EGTA (which results in 1.2 μM free Ca^2+^), 20 HEPES, 20 glucose, 1 ATP. For solutions with 3 μM and 10 μM free internal Ca^2+^ 1 mM HEDTA was used as Ca^2+^ buffer; 0.043 mM total Ca^2+^ (CaCl_2_) result in 3 μM free Ca^2+^, and 0.125 mM total Ca^2+^ result in 10 μM free Ca^2+^. Free internal Ca^2+^ concentrations were calculated with the Webmaxc extended calculator: http://www.stanford.edu/~cpatton/webmaxc/webmaxcE.htm. pH-values of all solutions were adjusted to 7.2. Osmolarities of solutions were controlled with a manually operated Micro-Osmometer (Type OM 806, Löser, Berlin, Germany) and adjusted to 315–325 mOsm. Within an experimental setting the difference in osmolarities did not exceed 5 mOsm.

ACA (Sigma, Vienna, Austria) was diluted into the standard bath solution (extracellular side of the cell membrane) to result in final bath concentrations of 300 μM, 1 mM, 3 mM, or 10 mM, into EtOH containing solutions (applied to the extracellular side of the cell membrane) to give a final concentration of 100 μM or into the standard pipette solution (intracellular side of the cell membrane) to produce final pipette concentrations of 30 nM, 100 nM, 300 nM, 1 μM, 30 μM, 100 μM, or 300 μM. Actual concentrations of ACA in solutions used for perfusion to the extracellular side were tested with an ACA-assay-kit (Megazyme, Bray, Ireland) according the manufactures guidelines. Since up to 20% of ACA concentrations were found to evaporate during 30 min at room temperature the effective concentrations were between 80–100 μM, usually close to 90 μM within the experimental time course. ACA containing solutions were discarded after 30 min. ACA and other equipment such as pipettes and pipette tips were kept in the fridge. Stock solutions were kept on ice and sealed with parafilm. All ACA containing solutions for perfusion were prepared immediately before use. Pipette solutions containing ACA held in the filling syringe were kept on ice and filled into the electrodes stored at room temperature. Then the electrodes were slightly warmed up by rubbing between the fingertips for at least 15 s and subsequently used for recordings. In order to rule out an impact of temperature itself on channel activity this procedure was also applied to control measurements.

### Electrophysiology

Recordings were performed at room temperature (20–23°C). Single channel recordings were obtained in the outside-out and inside-out mode as described previously by Sitdikova et al. (2010). Cell free patches were clamped to a holding potential of +30 mV for outside-out and −30 mV for inside-out patches which is caused by the sign inversion technically necessary in inside out patches to receive a +30 mV depolarization at the internal side of the membrane. All recordings were started with control perfusion (bath or pipette solution) over a period of 1 min in order to exclude false results introduced by sheer forces due to the flow of the perfusate. Microelectrodes were vertically pulled from borosilicate glass capillaries (GB150F-10, Science Products, Hofheim, Germany) for outside-out patches and from Garner Glass, Type 7052 (Claremont, California, USA) for inside-out patches. Patch pipettes used had usually tip resistances of 5–8 MegaOhm. Test solutions were applied via a gravity-driven perfusion system (ALA Scientific Instruments Westbury, New York, USA). For rapid solution exchange (about 300–500 ms) membrane patches were held in a stream of the experimental solution from a second pipette. Analog signals were amplified with an Axopatch 200B amplifier (Axon Instruments/ Molecular Devices, Sunnyvale, California, USA) and converted to digital signals by an Axon Instruments 1322A Digidata interface. Recordings were taken with a low pass Bessel filter at a frequency of 5 kHz and filtered offline at 1 kHz before further analyses using Axon pClamp10 software (Clampfit, Axon Instruments). Channel *Po* was expressed as *P*_open_ = *NPo*/*n*, where *NPo* = [(*t*_*o*_)/(*t*_*o*_ + *t*_*c*_)], *Po* = open probability for one channel, *t*_*o*_ = sum of open times, *t*_*c*_ = sum of closed times, *N* = actual number of channels in the patch, and *n* = maximum number of individual channels observed in the patch at +30 mV. Channel mean open time and unitary current amplitudes were measured using Clampfit software (Axon Instruments).

### Statistics

Measurements were replicated several times with different membrane patches. The number of recordings (i.e., “replicates” or *n*) per experiment is mentioned in the text or in the legends of the graphs. Each recording or n represents a single patch of an individual cell. Data are shown as arithmetic mean ± standard error of mean (SEM). Since original data partially exhibit non-normality and heteroscedasticity, the respective data sets were subjected to appropriate transformations [logit-transformation for *Po* and log-transformation for MCOT] before parametric statistical testing was applied. For statistical analyses the following parametric tests were then performed on the transformed data: Paired or unpaired Student's *t*-test, One-way or Repeated Measures ANOVA followed by Bonfferoni-corrected *post-hoc* tests. Statistic significance was assumed at a *p*-value of <0.05. Dose-response-relation was fitted with GraphPad Prism (GraphPad Software Inc., San Diego, USA) to the following sigmoidal dose-response-equation: *Y* = 1/(1 + 10^((LogEC50-X) × HillSlope)). *X* is the logarithm of concentration, *Y* is the response, EC50 is the half maximal effective concentration.

## Results

### Extracellular ACA

Extracellular ACA did not affect BK channel properties irrespective of the concentration applied [at free internal Ca^2+^ concentrations ([Ca^2+^]i) of 1.2 μM]. Data of all experiments were analyzed with regard to ACA mediated alterations in BK channel *Po* (Table 1), mean channel amplitude and MCOT (data not shown).

**Table 1:** **Effect of extracellular ACA (ACAe) on BK channel open probability (*Po*) compared to control conditions (con) at 1.2 μM [Ca^2+^]i and after wash out (w. o.)**.

**[ACA]e**	***Po* con**	***Po* ACAe**	***Po* w. o.**
300 μM (*n* = 7)	0.056 ± 0.015	0.059 ± 0.017	0.060 ± 0.020
1 mM (*n* = 9)	0.046 ± 0.017	0.048 ± 0.019	0.041 ± 0.010
3 mM (*n* = 6)	0.039 ± 0.005	0.043 ± 0.005	0.041 ± 0.005
10 mM (*n* = 6)	0.079 ± 0.043	0.072 ± 0.039	0.066 ± 0.035

Extracellular ACA in different concentrations ([ACA]e) did not affect BK channel open probability.

### Intracellular ACA

In single channel recordings from excised inside-out patches, ACA (100 μM) was applied to the intracellular side of the membrane. The effect of ACA was tested at ([Ca^2+^]i) of 1.2 μM (*n* = 10), 3 μM (*n* = 6), and 10 μM (*n* = 9). BK channel *Po* and single channel amplitudes were not affected by internal ACA irrespective of the [Ca^2+^]i (data not shown). However, mean open time of BK channels was significantly reduced at 1.2 μM [Ca^2+^]i (control: 1.931 ± 0.507 ms; ACA: 1.721 ± 0.546 ms^*^, Paired Student's *t*-test: ^*^p < 0.05), but not at 3 μM or 10 μM [Ca^2+^]i.

### Effect of ethanol on BK channels

**Figure 1:**
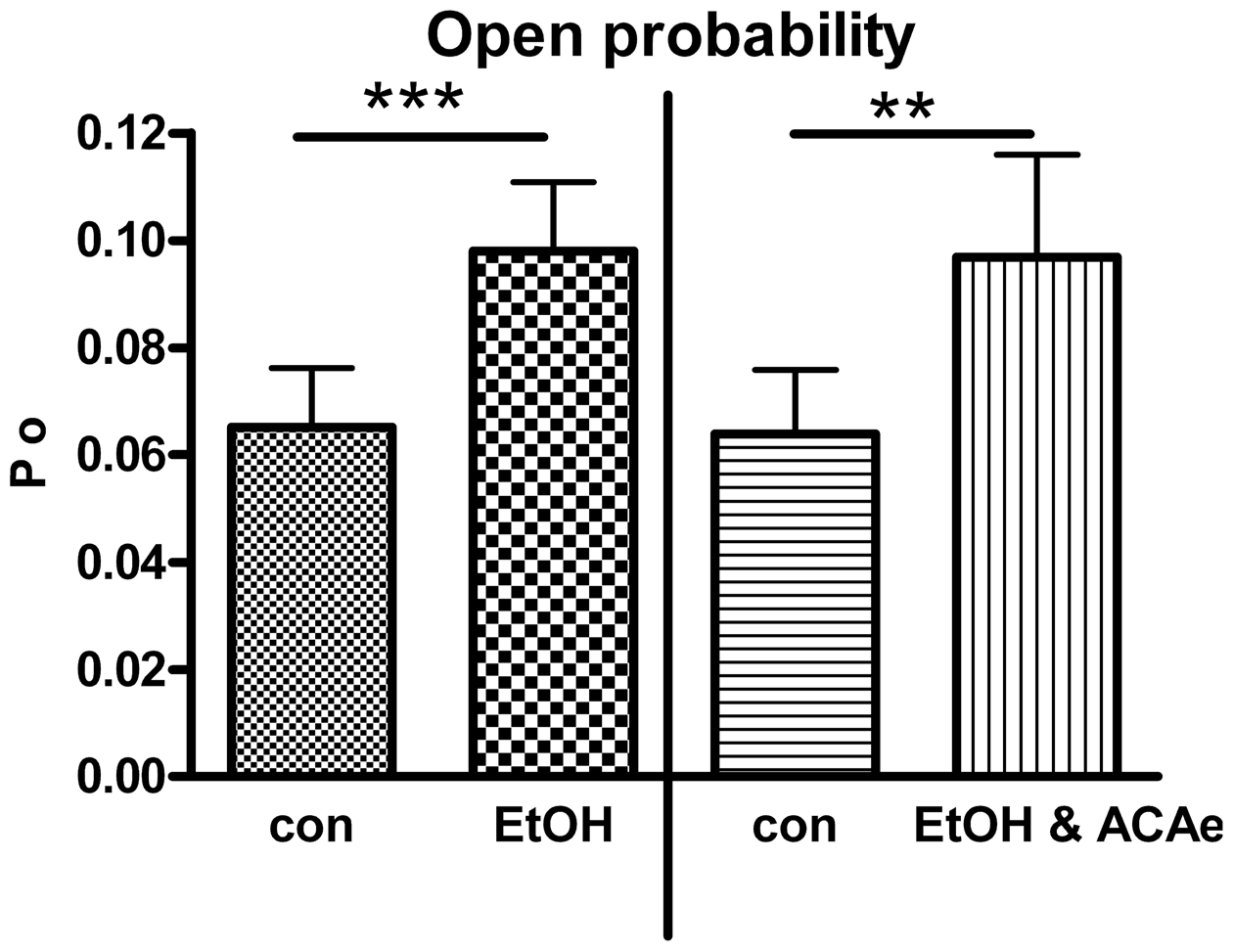
**Combined extracellular EtOH/ACA application**. Effect of 30 mM EtOH in absence (left panel, Paired Student's *t*-test: ^***^p < 0.001, *n* = 23) and in presence of ACA at the extracellular side (ACAe, right panel, Paired Student's *t*-test: ^**^p < 0.01, *n* = 16). In both cases open probability (*Po*) was significantly increased compared to control (con).

**Figure 2:**
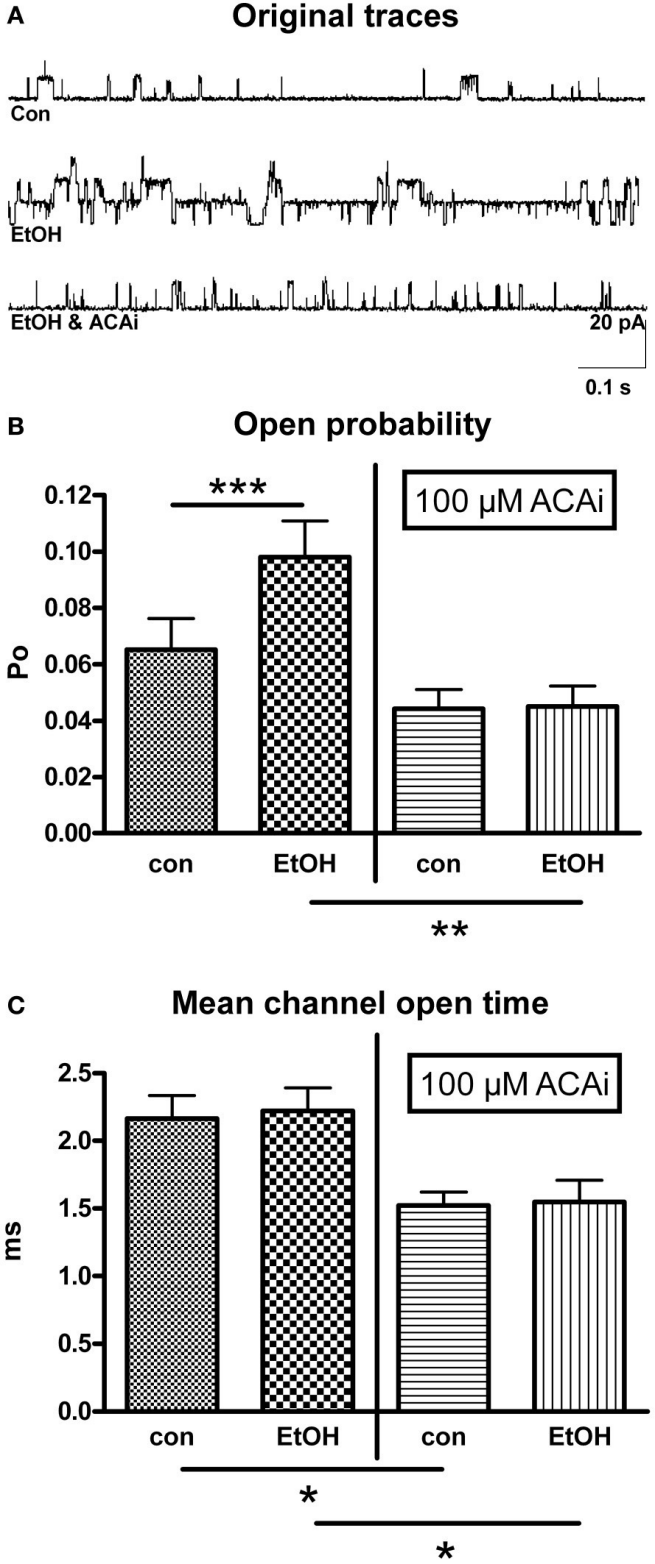
**Effect of EtOH in absence and presence of internal ACA (ACAi). (A)** Representative original traces from outside-out patches under control (con) conditions, during perfusion with EtOH alone and under the influence of EtOH and ACAi. **(B)** Open probability (*Po*) was significantly increased (*n* = 23, Paired Student's *t*-test: ^***^p < 0.001) by 30 mM EtOH compared to control (con, left panel). This increment was totally abolished in the presence of 100 μM ACAi (right panel, *n* = 13, Unpaired Student's *t*-test: ^**^p < 0.01). **(C)** Mean channel open time was significantly reduced in the presence of 100 μM ACAi (Unpaired Student's *t*-test: ^*^p < 0.05) under control conditions as well as under EtOH.

The effect of EtOH was tested at different [Ca^2+^]i of 1.2 μM, 3 μM, and 10 μM. Application of 30 mM isoosmolar EtOH increased BK channel *Po* significantly at low, but not at high [Ca^2+^]i (Table 2; also see Figures 1, 2B/left panels, respectively). The activation remained constant for the time of EtOH application (1 min) and was not transient as described previously by Jakab et al. (1997). Channel amplitudes and MCOTs were not affected (data not shown). The activating effect was fully reversible by reperfusion with bath solution.

**Table 2:** **Effect of 30 mM EtOH on BK channel open probability (*Po*) compared to control conditions (con) at different levels of [Ca^2+^]i**.

**[Ca^2^+]i**	***Po* con**	***Po* EtOH**	***Po* w. o.**
1.2 μM (*n* = 23)	0.065 ± 0.011	0.097 ± 0.012^***^	0.069 ± 0.011
3 μM (*n* = 9)	0.102 ± 0.023	0.120 ± 0.026^**^	0.101 ± 0.032
10 μM (*n* = 8)	0.246 ± 0.072	0.238 ± 0.063	0.207 ± 0.074

The increasing effect was fully reversible after wash out (w. o.). Paired Student's t-test:

*** p < *0.001*,

** p < *0.01*.

### Combined effect of EtOH and ACA

#### Extracellular ACA application

In outside-out single channel recordings EtOH (30 mM) was applied simultaneously in combination with ACA (100 μM) to the extracellular side of cell membrane at 1.2 μM [Ca^2+^]i. These experiments were done in order to reveal possible interactions of EtOH and ACA on BK channels at the outer surface of the membrane. BK channel *Po* was significantly increased by extracellular application of EtOH and ACA in combination. The EtOH-mediated increment of BK channel activity was highly significant irrespective of the presence of external ACA (Figure 1). Hence, external ACA was not able to affect EtOH action on BK channel activity. Amplitude and MCOT were not modified (data not shown).

#### Intracellular ACA application

In this experimental setting 30 mM isoosmolar EtOH was applied to outside-out patches via perfusion from the extracellular side. ACA was applied to the intracellular side of the membrane in a concentration of 100 μM by addition to the pipette solution. Therefore, internal ACA was present during the entire time course of the experiment. Figure 2A shows original traces from outside-out recordings under different experimental conditions corresponding to the bars in graph 2B. The increase of BK channel *Po* by extracellular EtOH, as shown in Figure 2B (left panel) was totally abolished by the simultaneous presence of 100 μM ACA at the intracellular side at 1.2 μM [Ca^2+^]i (Figure 2B/right panel). Furthermore, MCOT was significantly diminished in the presence of internal ACA (Figure 2C), but mean channel amplitudes were not affected.

**Figure 3:**
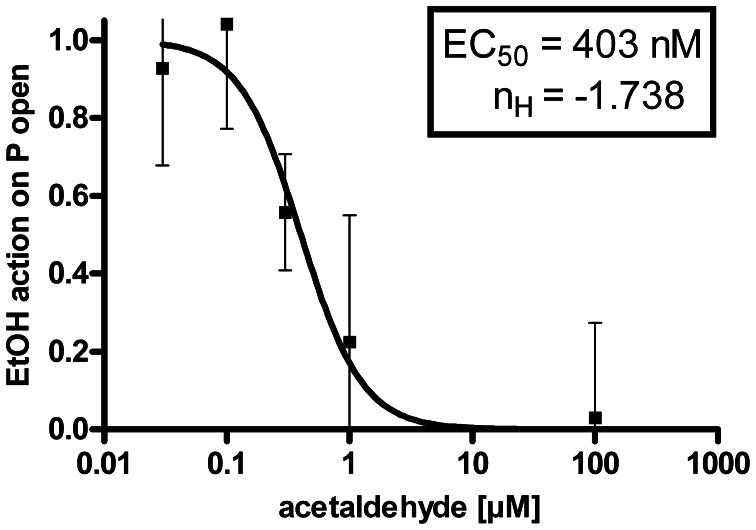
**Dose-response-relationship:** The EtOH effect on BK channel open probability (P open) was progressively inhibited by increasing concentrations of ACA at the intracellular side of the membrane (EC_50_ = half maximal effective concentration = 403 ± 108 nM, *n*_*H*_ = Hill coefficient = −1.738 ± 0.731, semilogarithmic graph).

The suppression of the EtOH-mediated increment of BK channel activity by internal ACA was dose dependent. Experiments were performed at 1.2 μM [Ca^2+^]i and intracellular ACA concentrations were increased from 30 nM (*n* = 10), 100 nM (*n* = 6), 300 nM (*n* = 6), 1 μM (*n* = 6), 30 μM (*n* = 7), 100 μM (*n* = 13) to 300 μM (*n* = 6). Figure 3 shows a dose–response relationship with an EC50 at 403 nM ACA and a Hill coefficient (*n*_*H*_) of −1.738. The augmentation of BK channel *Po* at 30 mM isoosmolar EtOH as reference was set to 1.

### EtOH and ACA application in variable succession

**Figure 4:**
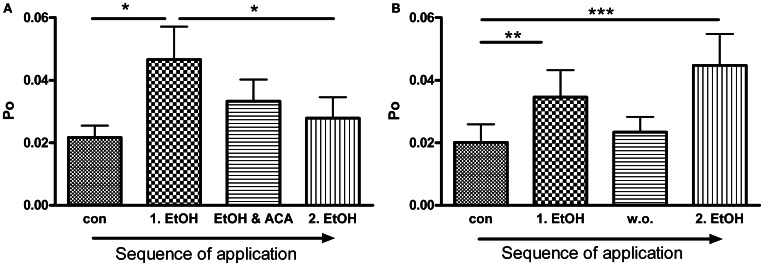
**ACA inhibits further EtOH action: (A)** BK channel open probability (*Po*) was significantly increased by the first EtOH application. The following simultaneous treatment with ACA and EtOH reduced the EtOH induced increment and prevented activation by a 2^nd^ separate EtOH (2.EtOH) application (*n* = 11, con vs. 1.EtOH ^*^p < 0.05, 1.EtOH vs. 2.EtOH ^*^p < 0.05, Repeated Measures ANOVA followed by Bonferroni's Multiple Comparison Test). **(B)** In control experiments a 2^nd^ separate EtOH (2.EtOH) application after perfusion with control solution (1 min, wash out, w. o.) increased BK channel activity significantly. Without a preceding ACA application EtOH mediated activation was not impeded (outside-out patches, *n* = 9, con vs. 1.EtOH ^**^p < 0.01, con vs. 2.EtOH ^***^p < 0.001, Repeated Measures ANOVA followed by Bonferroni's Multiple Comparison Test).

In further experiments we tested if there is any difference in the interaction of EtOH and ACA in dependency of succession of the drugs. Our results indicate a “first come, first serve” effect. In inside-out experiments 30 mM EtOH and/or 100 μM ACA were applied to the intracellular side of the membrane in varying sequences. Recordings were performed at 1.2 μM [Ca^2+^]i. Original data of the individual experiments as well as the sequence of application are listed in Tables 3, 4 and Figure 4, respectively.

**Table 3:**
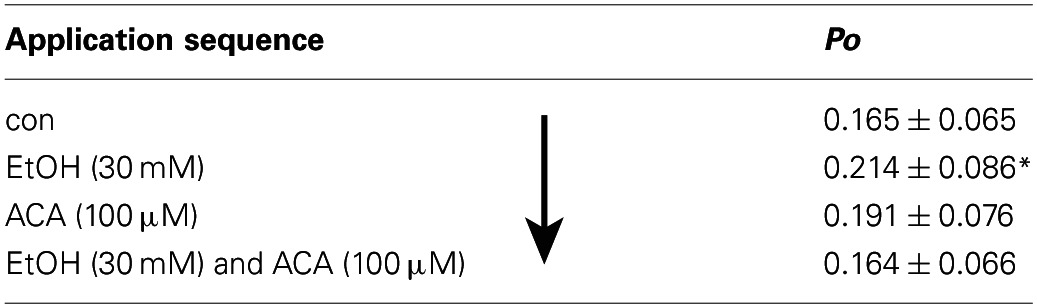
**Sequence (↓) of application from top to bottom: con–EtOH–ACA–EtOH and ACA together**.

Only the application of EtOH alone resulted in a significant increase of BK channel open probability (Po), (n = 5, Repeated Measures ANOVA followed by Bonferroni's Multiple Comparison Test: ^*^p < *0.05*) compared to control (con).

**Table 4:**
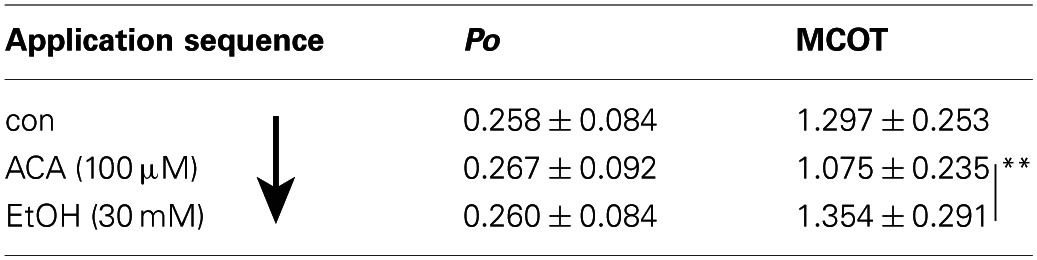
**Sequence (↓) of application from top to bottom: con–ACA–EtOH**.

BK channel open probability (Po) was affected neither by ACA nor by EtOH (n = 6, Repeated Measures ANOVA followed by Bonferroni's Multiple Comparison Test), but mean channel open time (MCOT) was significantly lower under ACA treatment compared to MCOT under EtOH (ACA vs. EtOH, ^**^p < *0.01*, Paired t-test).

### ACA succeeds an EtOH application

As known from previous experiments (see also Table 2) BK channel *Po* was increased by EtOH when applied subsequent to a control solution. The following ACA reduced EtOH action. During ensuing application of EtOH and ACA in combination BK channel activity recovered to control level. Table 3 displays the sequence of application in direction of the arrow. The data show that the EtOH effect on BK channel *Po* is prevented following an ACA application and in presence of internal ACA, respectively. Channel amplitudes and mean open times were not affected (data not shown).

### EtOH succeeds ACA application

In this experimental setting the order was reversed, i.e., EtOH was applied following an ACA application. Both substances were applied separately. Internal ACA did not change BK channel *Po* significantly (as already described above), but surprisingly the action of a following EtOH application was inhibited regardless of the absence of ACA (Table 4). This indicates that the prevention of an EtOH-mediated increment on the *Po* was a lasting effect that occurred also after removal of ACA within the experimental time of 30 s after switching from ACA to EtOH. Channel amplitudes were influenced neither by EtOH nor by ACA, but MCOT was significantly lower under the impact of ACA compared to EtOH conditions.

### Permanent presence of EtOH

BK channel *Po* was significantly increased by the first EtOH application. In the presence of ACA this EtOH-mediated increment was progressively reduced and abolished, even when ACA was removed. A second application of EtOH alone was not able to activate BK channels anymore (Figure 4A). In control experiments we could show that EtOH is well able, however, to cause BK channel activation a second time, after a 1 min wash out with control solution (Figure 4B). Inhibition of another EtOH action did not arise without preceding ACA application. In both experimental settings channel amplitudes and MCOTs were not affected (data not shown).

### Effect of ACA on hypotonicity induced BK channel activation

**Figure 5:**
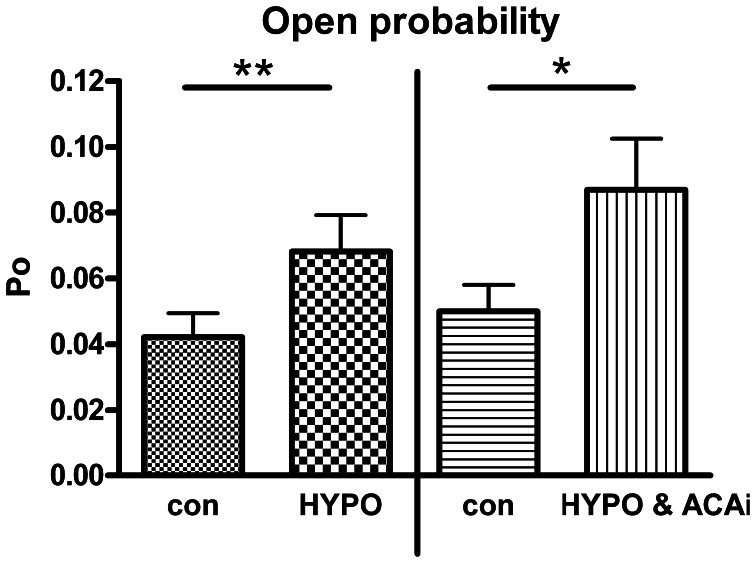
**Effect of internal ACA on hypotonicity induced BK channel activation**. BK channel open probability was significantly increased by a hypotonic solution at 1.2 μM [Ca^2+^]i (left panel, Paired Student's *t*-test: ^**^p < 0.01, *n* = 4). Internal ACA (ACAi) did not affect this hypotonicity induced activation (right panel, Paired Student's *t*-test: ^*^p < 0.05, *n* = 12).

Beside EtOH, Hypo is also well known to mediate BK channel activation (Jakab et al., 2006). To investigate whether ACA specifically modulated EtOH-induced BK channel activation or, rather, ACA modulatory action extended to other BK channel activators, we tested the effect of internal ACA on Hypo-induced BK channel activation in outside-out recordings. A 30% hypotonic solution increased BK channel activity significantly. Internal ACA (100 μM) was not able to modify this increasing effect (Figure 5).

## Discussion

ACA is supposed to be responsible for some of the pharmacological and neurobehavioral effects which so far have been assigned to EtOH (Quertemont et al., 2005a,b). We focused our investigation on the direct ACA-mediated effects on BK channels as well as on the interference of ACA and EtOH. EtOH increases BK channel *Po* (Dopico et al., 1996; Jakab et al., 1997). ACA, as the first metabolic EtOH product, occurs concurrently with its progenitor under physiological conditions and hence we hypothesized that the simultaneous application of both substances may cause interactions.

Extracellular application of ACA and EtOH represents a physiological situation which occurs when these molecules diffuse from the vascular system to the cells within the brain. EtOH as an amphiphilic molecule is able to penetrate the blood brain barrier (BBB) (Mendelson et al., 1990), to pass the lipid phase of cell membranes and to diffuse within the cytosol. ACA should at least partly be able to pass the BBB by diffusion (Quertemont et al., 2005a; Correa et al., 2011). However, since aldehyde dehydrogenase (ALDH) is highly effective in endothelial cells of the BBB, the amount of ACA in the brain produced by peripheral EtOH metabolism is rather small (Deitrich, 1987; Zimatkin, 1991). Hence, ACA concentrations derived from blood circulation are thought to be insufficient to cause central effects within the brain (Zimatkin et al., 2006; Zimatkin and Buben, 2007). Recent research indicates that both central EtOH degradation by catalase and peripherally produced ACA contribute to ACA accumulation in the brain (Jamal et al., 2007). We therefore tested if extracellular application of ACA influences BK channels. Concentrations of up to 10 mM did not affect BK channel properties. We also applied EtOH and ACA simultaneously to the extracellular side of the channels, but ACA did not alter the augmented channel activity induced by the action of EtOH. Since internally applied ACA, as discussed below, reduced BK channel mean open time significantly, these experiments support the idea that ACA is not able to cross the cell membrane in the short time range of a few minutes.

After alcohol consumption ACA and EtOH are present together in the body. However, the concurrent application of EtOH and ACA has not been investigated previously. Concerning the simultaneous existence of EtOH and ACA in the brain as a consequence of EtOH degradation it was shown that EtOH oxidation occurs in the living brain. A study by Zimatkin et al. (2006) confirmed that catalase, an enzyme which predominantly occurs in peroxisomes, plays a major role in the brain EtOH metabolism. The finding of ACA accumulation within cells (Zimatkin et al., 2006; Zimatkin and Buben, 2007) led to the conclusion that ACA can achieve some of its effects from the intracellular side of the membrane. Our experiments show that intracellular ACA prevents the EtOH mediated increment of BK channel activity. This inhibition of the EtOH action on the BK channels was dose-dependent. The inhibitory impact of ACA on EtOH-induced BK channel activation did not change single channel conductance which indicates that ACA does not interfere with potassium ions passing through the channel, neither does EtOH affect this ion passage (Brodie et al., 2007; Treistman and Martin, 2009). MCOT was reduced in the presence of internal ACA which points to an interaction of ACA with the channel gating process. The reduction of MCOT by ACA was observed when applied together with EtOH, but also when ACA was applied separately, suggesting a direct, non-EtOH dependent interaction of ACA with BK channels. In all our studies EtOH was unable to activate BK channels after a preceding internal ACA application, and most notably, the prevention of EtOH activation was a lasting effect which persisted after ACA removal, i.e., the continued presence of ACA was not mandatory. On the other hand, if EtOH was applied previous to ACA, it was not able to sustain its effect on BK channels. The EtOH-mediated increment of BK channel *Po* was rapidly reduced and finally abolished in spite of the continued presence of EtOH. In this respect EtOH and ACA appear to obey a “first come, first serve” rule, since ACA was able to counteract the action of acute EtOH on BK channels in a lasting way when applied first.

BK channels are known to play a key role in behavioral tolerance to EtOH, since BK loss-of-function mutants of *C. elegans* are resistant to EtOH (Davies et al., 2003). Furthermore, Cowmeadow et al. (2005) could show in *D. melanogaster* that EtOH tolerance was only observed when BK channels were expressed. In BK null flies the capacity for tolerance was eliminated. Tolerance develops as a consequence of prolonged or repeated drug consumption. This raises the question whether ACA may contribute to the mechanism(s) causing tolerance. In fact, BK channels display tolerance to EtOH-mediated effects after short- or long-term exposure which is manifested by a decrease in BK channel potentiation under continuous or repeated EtOH exposure (Jakab et al., 1997; Pietrzykowski et al., 2004; Yuan et al., 2008). This so-called molecular tolerance is intrinsic to BK channel alpha (α)-subunits and appears in the form of reduced sensitivity to EtOH within a few minutes due to a decrease in Ca^2+^ sensitivity during persistent exposure (Feinberg-Zadek et al., 2008). In presence of the accessory and modulatory β4-subunit tolerance disappears (Martin et al., 2008). The lipid environment is an additional crucial factor modulating intrinsic tolerance of BK channel α-subunits (Yuan et al., 2008). Physiologically activation of BK channels by EtOH alters action potential discharge activity and neurotransmitter release. Since the cell tries to countervail against these alterations in order to keep the system in balance, these perturbations on the molecular level may have powerful influence on behavioral tolerance and addiction (Treistman and Martin, 2009). As the reduction of sensitivity to EtOH is a considerable component of tolerance ACA could be involved in this process. It could be argued that the inhibitory impact of internal ACA on EtOH related augmentation of BK channel activity reflects a kind of ‘protective’ effect under acute EtOH exposure, maintaining neuronal activity and excitability. This is interesting with regard to the continued ACA action which is preserved also after its removal.

Our study shows that internal ACA reduces MCOT under control conditions as well as in presence of EtOH. This reduction of MCOT did not result in alterations of *Po*, which can be explained by more frequent channel openings. We interpret this result as evidence of an interaction of ACA with the BK channel gating mechanism. It remains to be investigated if this is a direct effect where ACA interacts with some site of the channel protein, or is an indirect effect via some signaling pathways, such as phosphorylation. There is evidence that the EtOH-related activation of BK channels is due to the stimulation of PKC (Jakab et al., 1997) indicating that phosphorylation is an efficient modulatory factor in this process (Liu et al., 2006). Therefore, the inhibition of the EtOH-related effect via ACA could be caused by prevention of PKC-mediated phosphorylation. The mechanisms of ACA engagement in PKC phosphorylation processes need further investigation.

Since the functional efficiency of some PKC species relies on intracellular Ca^2+^ availability ACA could achieve its counteracting effect on EtOH-induced BK channel activation by engaging with the Ca^2+^ influx into the cytosol. These considerations agree with the finding that ACA inhibits voltage-dependent Ca^2+^ channels. The inhibition of L-type Ca^2+^ channels by ACA was demonstrated both in neurons (Bergamaschi et al., 1988) and smooth muscle cells (Morales et al., 1997). Liu et al. (2008) postulate that EtOH may simply act as an adjuvant of activating Ca^2+^ by selectively facilitating Ca^2+^-driven gating, but without triggering alterations in protein conformation of BK channels or rearrangement of subunits. In addition, EtOH was shown to fail its activating action on BK channels in the absence of Ca^2+^. In fact, EtOH activation of BK channels depends on the amount of internally present Ca^2+^, displaying potentiation only at low but not at high Ca^2+^ concentrations (Dopico et al., 1998). Our results in this study confirm these findings. The effectiveness of the Ca^2+^ action depends on the high affinity sensors within the intracellular BK channel tail of the α-subunit, namely the calcium bowl and the RCK1 (regulatory domain of K conductance). However, the RCK 1 domain is sufficient to promote inhibition at high Ca^2+^ levels. Hence, very high internal Ca^2+^ concentrations have a toxic impact on the physiological state of the cell, since inhibition of BK channels implies a lack of protection from excitotoxicity (Liu et al., 2008). The findings of our study demonstrate that internal ACA at high nanomolar concentrations is able to counteract BK channel potentiation by EtOH at low Ca^2+^ levels. At high Ca^2+^ levels ACA did not exhibit any decreasing effect on BK channel activity or MCOT, indicating that ACA is not able to override the impact of high Ca^2+^.

Beside EtOH, Hypo is another mechanism which leads to an increase of BK channel activity (Jakab et al., 2006). The presence of intracellular ACA did not prevent the activation of BK channels by Hypo. These findings suggest that EtOH and Hypo affect BK channels by different mechanisms and implicate a specific interaction of ACA and EtOH on BK channels.

The reasons for the stunning absence of basic knowledge concerning effects of ACA on ion channels may be due to its chemical and physical characteristics. ACA is highly volatile at room temperature which complicates the application of ACA in experiments performed especially *in vitro*. A further problem is that ACA concentrations of both blood and brain are difficult to quantify since the techniques to measure ACA levels by brain micro-dialysis *in vivo* is limited. Moreover, *in vivo* administered ACA is rapidly converted to EtOH by alcohol dehydrogenase (ADH) in the liver and to acetate by ALDH in the liver and in the brain. In addition the question whether significant ACA concentrations accumulate in the brain after alcohol ingestion is still a topic of controversial discussions (Deng and Deitrich, 2008; Correa et al., 2011). In consideration of these experimental and methodical restrictions it is not surprising that research on ACA is difficult and may still lead to inconsistent results.

In summary, our study supports the notion that ACA is a key player in the context of EtOH action. ACA achieves its immediate effects on BK channels only from the intracellular side of the membrane. Furthermore, ACA does not interfere with BK channel activation by Hypo. This evidence suggests that EtOH, ACA, and Hypo affect BK channels via different mechanisms. The inhibitory impact of ACA on the EtOH mediated increase of BK channel activity implicates that ACA has to be carefully taken into account if EtOH effects are studied. ACA and EtOH should be treated as an entity in the context of the EtOH action, whose compound effects may be more dramatic than those of the individual drugs.

### Conflict of interest statement

The authors declare that the research was conducted in the absence of any commercial or financial relationships that could be construed as a potential conflict of interest.

## References

[B1] Bergamaschi S. Govoni S. Rius R. A. Trabucchi M. (1988). Acute ethanol and acetaldehyde administration produce similar effects on L-type calcium channels in rat brain. Alcohol 5, 337–340 10.1016/0741-8329(88)90076-6 2852497

[B2] Brodie M. S. Scholz A. Weiger T. M. Dopico A. M. (2007). Ethanol interactions with calcium-dependent potassium channels. Alcohol. Clin. Exp. Res. 31, 1625–1632 (Review). 10.1111/j.1530-0277.2007.00469.x 17850640

[B3] Chatterjee O. Taylor L. A. Ahmed S. Nagaraj S. Hall J. J. Finckbeiner S. M. (2009). Social stress alters expression of large conductance calcium-activated potassium channel subunits in mouse adrenal medulla and pituitary glands. J. Neuoendocrinol. 21, 167–176 10.1111/j.1365-2826.2009.01823.x 19207824

[B4] Church J. Baxter K. A. McLarnon J. G. (1998). pH modulation of Ca^2+^ responses and a Ca^2+^-dependent K^+^ channel in cultured rat hippocampal neurons. J. Physiol. 511(Pt 1), 119–132 10.1111/j.1469-7793.1998.119bi.x 9679168 PMC2231090

[B5] Correa M. Salamone J. D. Segovia K. N. Pardo M. Longoni R. Spina L. (2011). Piecing together the puzzle of acetaldehyde as a neuroactive agent. Neurosci. Biobehav. Rev. 36, 404–430 10.1016/j.neubiorev.2011.07.009 21824493

[B6] Cowmeadow R. B. Krishnan H. R. Atkinson N. S. (2005). The slowpoke gene is necessary for rapid ethanol tolerance in Drosophila. Alcohol. Clin. Exp. Res. 29, 1777–1786 10.1097/01.alc.0000183232.56788.62 16269907

[B7] Davies A. G. Pierce-Shimomura J. T. Kim H. Vanhoven M. K. Thiele T. R. Bonci A. (2003). A central role of the BK potassium channel in behavioral responses to ethanol in *C. elegans.* Cell 115, 655–666 10.1016/S0092-8674(03)00979-6 14675531

[B9] Deitrich R. A. (1987). Specificity of the action of ethanol in the central nervous system: behavioral effects. Alcohol Alcohol. Suppl. 1, 133–138 3322305

[B10] Deng X. S. Deitrich R. A. (2008). Putative role of brain acetaldehyde in ethanol addiction. Curr. Drug Abuse Rev. 1, 3–8 10.2174/1874473710801010003 19122804 PMC2613359

[B8] DiChiara T. J. Reinhart P. H. (1997). Redox modulation of hslo Ca^2+^-activated K^+^ channels. J. Neurosci. 17, 4942–4955 9185532 10.1523/JNEUROSCI.17-13-04942.1997 PMC6573296

[B11] Dopico A. M. Anantharam V. Treistman S. N. (1998). Ethanol increases the activity of Ca(++)-dependent K+ (mslo) channels: functional interaction with cytosolic Ca++. J. Pharmacol. Exp. Ther. 284, 258–268 9435186

[B12] Dopico A. M. Chu B. Lemos J. R. Treistman S. N. (1999). Alcohol modulation of calcium-activated potassium channels. Neurochem. Int. 35, 103–106 10.1016/S0197-0186(99)00051-0 10405993

[B13] Dopico A. M. Lemos J. R. Treistman S. N. (1996). Ethanol increases the activity of large conductance, Ca^2+^-activated K^+^ channels in isolated neurohypophysial terminals. Mol. Pharmacol. 49, 40–48 8569710

[B14] Eriksson C. J. (2001). The role of acetaldehyde in the actions of alcohol (update 2000). Alcohol. Clin. Exp. Res. 25 5 Suppl. ISBRA, 15S–32S 10.1111/j.1530-0277.2001.tb02369.x 11391045

[B15] Feinberg-Zadek P. L. Martin G. Treistman S. N. (2008). BK channel subunit composition modulates molecular tolerance to ethanol. Alcohol. Clin. Exp. Res. 32, 1207–1216 10.1111/j.1530-0277.2008.00704.x 18537940

[B16] Foddai M. Dosia G. Spiga S. Diana M. (2004). Acetaldehyde increases dopaminergic neuronal activity in the VTA. Neuropsychopharmacology 29, 530–536 10.1038/sj.npp.1300326 14973432

[B17] Hermann A. Sitdikova G. F. Weiger T. M. (2012a). BK Channels—Focus on polyamines, ethanol/acetaldehyde and hydrogen sulfide (H2S), in Patch Clamp Technique, ed Kaneez F. S. (Rijeka: InTech), 109–142

[B17a] Hermann A. Sitdikova G. F. Weiger T. M. (2012b). Modulated by gasotransmitters: BK channels, in Gasotransmitters: Physiology and Pathophysiology, eds Hermann A. Sitdikova G. F. Weiger T. M. (Berlin, Heidelberg, New York: Springer), 163–201

[B18] Jakab M. Schmidt S. Grundbichler M. Paulmichl M. Hermann A. Weiger T. (2006). Hypotonicity and ethanol modulate BK channel activity and chloride currents in GH4/C1 pituitary tumour cells. Acta Physiol. (Oxf.) 187, 51–59 10.1111/j.1748-1716.2006.01544.x 16734742

[B19] Jakab M. Weiger T. M. Hermann A. (1997). Ethanol activates maxi Ca^2+^-activated K^+^ channels of clonal pituitary (GH3) cells. J. Membr. Biol. 157, 237–245 10.1007/PL00005895 9178611

[B20] Jamal M. Ameno K. Uekita I. Kumihashi M. Wang W. Ijiri I. (2007). Catalase mediates acetaldehyde formation in the striatum of free-moving rats. Neurotoxicol. 28, 1245–1248 10.1016/j.neuro.2007.05.002 17597213

[B21] Karahanian E. Quintanilla M. E. Tampier L. Rivera-Meza M. Bustamante D. Gonzalez-Lira V. (2011). Ethanol as a prodrug: brain metabolism of ethanol mediates its reinforcing effects. Alcohol. Clin. Exp. Res. 35, 606–612 10.1111/j.1530-0277.2011.01439.x 21332529 PMC3142559

[B22] Liu J. Asuncion-Chin M. Liu P. Dopico A. M. (2006). CaM kinase II phosphorylation of slo Thr107 regulates activity and ethanol responses of BK channels. Nat. Neurosci. 9, 41–49 10.1038/nn1602 16341213 PMC2574430

[B23] Liu J. Vaithianathan T. Manivannan K. Parrill A. Dopico A. M. (2008). Ethanol modulates BKCa channels by acting as an adjuvant of calcium. Mol. Pharmacol. 74, 628–640 10.1124/mol.108.048694 18552122 PMC2764333

[B24] Martin G. E. Hendrickson L. M. Penta K. L. Friesen R. M. Pietrzykowski A. Z. Tapper A. R. (2008). Identification of a BK channel auxiliary protein controlling molecular and behavioral tolerance to alcohol. Proc. Natl. Acad. Sci. U.S.A. 105, 17543–17548 10.1073/pnas.0801068105 18981408 PMC2582289

[B25] McManus O. B. (1991). Calcium-activated potassium channels: regulation by calcium. J. Bioenerg. Biomembr. 23, 537–560 1917908 10.1007/BF00785810

[B26] Melis M. Enrico P. Peana A. T. Diana M. (2007). Acetaldehyde mediates alcohol activation of the mesolimbic dopamine system. Eur. J. Neurosci. 26, 2824–2833 10.1111/j.1460-9568.2007.05887.x 18001279

[B27] Mendelson J. Woods B. T. Chiu T. M. Mello N. K. Lukas S. E. Teoh S. K. (1990). Measurement of brain ethanol concentrations in humans with *in vivo* proton magnetic resonance spectroscopy. NIDA Res. Monogr. 105, 68–74 1876151

[B28] Montgomery J. R. Whitt J. P. Wright B. N. Lai M. H. Meredith A. L. (2013). Mis-expression of the BK K(+) channel disrupts suprachiasmatic nucleus circuit rhythmicity and alters clock-controlled behavior. Am. J. Physiol. Cell Physiol. 304, C299–C311 10.1152/ajpcell.00302.2012 23174562 PMC3566534

[B29] Morales J. A. Ram J. L. Song J. Brown R. A. (1997). Acetaldehyde inhibits current through voltage-dependent calcium channels. Toxicol. Appl. Pharmacol. 143, 70–74 10.1006/taap.1996.8072 9073593

[B30] Pietrzykowski A. Z. Martin G. E. Puig S. I. Knott T. K. Lemos J. R. Treistman S. N. (2004). Alcohol tolerance in large-conductance, calcium-activated potassium channels of CNS terminals is intrinsic and includes two components: decreased ethanol potentiation and decreased channel density. J Neurosci. 24, 8322–8332 10.1523/JNEUROSCI.1536-04.2004 15385615 PMC6729695

[B31] Quertemont E. Tambour S. Tirelli E. (2005a). The role of acetaldehyde in the neurobehavioral effects of ethanol: a comprehensive review of animal studies. Progress Neurobiol. 75, 247–274 10.1016/j.pneurobio.2005.03.003 15882776

[B32] Quertemont E. Eriksson C. J. Zimatkin S. M. Pronko P. S. Diana M. Pisano M. (2005b). Is ethanol a pro-drug? Acetaldehyde contribution to brain ethanol effects. Alcohol. Clin. Exp. Res. 29, 1514–1521 16156048 10.1097/01.alc.0000175015.51329.45

[B33] Reinhart P. H. Chung S. Martin B. L. Brautigan D. L. Levitan I. B. (1991). Modulation of calcium-activated potassium channels from rat brain by protein kinase A and phosphatase 2A. J. Neurosci. 11, 61627–61635 1646298 10.1523/JNEUROSCI.11-06-01627.1991 PMC6575393

[B34] Reinhart P. H. Levitan I. B. (1995). Kinase and phosphatase activities intimately associated with a reconstituted calcium-dependent potassium channel. J. Neurosci. 15, 4572–4579 7790924 10.1523/JNEUROSCI.15-06-04572.1995 PMC6577735

[B35] Rodd-Henricks Z. A. Melendez R. I. Zaffaroni A. Goldstein A. McBride W. J. Li T. K. (2002). The reinforcing effects of acetaldehyde in the posterior ventral tegmental area of alcohol-preferring rats. Pharmacol. Biochem. Behav. 72, 55–64 10.1016/S0091-3057(01)00733-X 11900769

[B36] Sitdikova G. F. Weiger T. M. Hermann A. (2010). Hydrogen sulfide increases calcium-activated potassium (BK) channel activity of rat pituitary tumor cells. Pflüg. Arch. 459, 389–397 10.1007/s00424-009-0737-0 19802723

[B37] Tashjian A. H. Jr. Bancroft F. C. Levine L. (1970). Production of both prolactin and growth hormone by clonal strains of rat pituitary tumor cells. Differential effects of hydrocortisone and tissue extracts. J. Cell Biol. 47, 61–70 5513559 10.1083/jcb.47.1.61 PMC2108385

[B38] Treistman S. N. Martin G. E. (2009). BK Channels: mediators and models for alcohol tolerance. Trends Neurosci. 32, 629–637 10.1016/j.tins.2009.08.001 19781792 PMC4115799

[B39] Weiger T. M. Hermann A. (2009). Modulation of potassium channels by polyamines, in Biological Aspects of Biogenic Amines, Polyamines and Conjugates, ed Dandrifosse G. (Kerala: Transworld Research Network), 185–199

[B40] Weiger T. M. Holmqvist M. H. Levitan I. B. Clark F. T. Sprague S. Huang W. J. (2000). A novel nervous system beta subunit that downregulates human large conductance calcium-dependent potassium channels. J. Neurosci. 20, 3563–3570 10804197 10.1523/JNEUROSCI.20-10-03563.2000 PMC6772688

[B41] Yuan C. O'Connell R. J. Wilson A. Pietrzykowski A. Z. Treistman S. N. (2008). Acute alcohol tolerance is intrinsic to the BKCa protein, but is modulated by the lipid environment. J Biol. Chem. 283, 5090–5098 10.1074/jbc.M708214200 18084004 PMC4127471

[B42] Zimatkin S. M. (1991). Histochemical study of aldehyde dehydrogenase in the rat CNS. J. Neurochem. 56, 1–11 10.1111/j.1471-4159.1991.tb02555.x 1987314

[B43] Zimatkin S. M. Buben A. L. (2007). Ethanol oxidation in the living brain. Alcohol Alcohol. 42, 529–532 10.1093/alcalc/agm059 17660523

[B44] Zimatkin S. M. Liopo A. V. Deitrich R. A. (1998). Distribution and kinetics of ethanol metabolism in rat brain. Alcohol. Clin. Exp. Res. 22, 1623–1627 10.1111/j.1530-0277.1998.tb03958.x 9835273

[B45] Zimatkin S. M. Pronko S. P. Vasiliou V. Gonzalez F. J. Deitrich R. A. (2006). Enzymatic mechanisms of ethanol oxidation in the brain. Alcohol. Clin. Exp. Res. 30, 1500–1505 10.1111/j.1530-0277.2006.00181.x 16930212

